# Vector competence of Swedish *Culex pipiens* mosquitoes for Usutu virus

**DOI:** 10.1016/j.onehlt.2024.100707

**Published:** 2024-03-09

**Authors:** Janina Krambrich, Emma Bole-Feysot, Patrick Höller, Åke Lundkvist, Jenny C. Hesson

**Affiliations:** a Zoonosis Science Center, Department of Medical Biochemistry and Microbiology, Uppsala University, Uppsala, Sweden; b Biologisk Myggkontroll, Nedre Dalälvens Utvecklings AB, Gysinge, Sweden

**Keywords:** Flavivirus, *Culex pipiens*, Mosquito, Usutu virus, Vector competence, USUV plaque assay

## Abstract

Usutu virus (USUV) is an emerging mosquito-borne flavivirus with increasing prevalence in Europe. Understanding the role of mosquito species in USUV transmission is crucial for predicting and controlling potential outbreaks. This study aimed to assess the vector competence of Swedish *Culex pipiens* for USUV. The mosquitoes were orally infected with an Italian strain of USUV (Bologna 2009) and infection rates (IR), dissemination rates (DR), and transmission rates (TR) were evaluated over 7 to 28 days post-infection. The study revealed that Swedish *Cx. pipiens* are susceptible to USUV infection, with a gradual decrease in IR over time. However, the percentage of mosquitoes with the ability to transmit the virus remained consistent across all time points, indicating a relatively short extrinsic incubation period. Overall, this research highlights the potential of Swedish *Cx. pipiens* as vectors for USUV and emphasizes the importance of surveillance and monitoring to prevent future outbreaks of mosquito-borne diseases.

## 1 Introduction

Usutu virus (USUV) is an emerging mosquito-borne flavivirus from the Japanese encephalitis virus antigenic complex that belongs to the genus *Flavivirus* of the family *Flaviviridae* [1]. It is a positive single-stranded RNA virus with a genome length of approximately 11,000 nucleotides that encodes for a poly protein which is cleaved into three structural and seven non-structural proteins [2]. USUV is closely related to Japanese encephalitis virus (JEV). It is transmitted primarily by mosquitoes of the genus *Culex*, with birds serving as amplifying hosts [3,4]. Seroprevalence studies suggest that USUV infections in humans are mostly asymptomatic and the frequency of such cases is currently unknown [5]. Symptomatic human infections typically involve mild fever, occasionally with arthralgia, myalgia, rash or jaundice [[6], [7], [8]]. Initial cases were observed in Africa, with the first patients reported from the Central African Republic and Burkina Faso, presenting with fever and rash [9]. Subsequent human cases have emerged primarily in Europe. Despite evidence of active virus circulation in Africa from seroprevalence studies no further human disease cases have been reported there [10,11].

USUV was first isolated in 1959 from *Culex neavei* found near the Usutu River in South Africa and has subsequently been detected in birds and *Culex* mosquitoes in sub-Saharan countries [[12], [13], [14], [15], [16], [17]]. A retrospective study of Eurasian blackbirds (*Turdus merula*) from Italy in 1996 was the first documented evidence of an introduction of USUV into Europe [18,19]. The virus was next detected in Austria in 2001, where it caused high mortality in blackbirds [20]. Since then, USUV has spread to many European countries, including the Czech Republic [21], Germany [22,23], Hungary [24], Spain [25], Switzerland [26], United Kingdom [27], Poland [28], Croatia [29,30], and the Netherlands in 2016 [31], where it again caused high mortalities in birds, but now in both blackbirds and captive great grey owls (*Strix nebulosa*). Migratory birds are thought to be responsible for spreading the virus in Africa and Europe, both over short and long distances [32,33].

Surveillance efforts of European mosquito species have detected USUV primarily in the northern house mosquito, *Cx. pipiens*, one of the most abundant species in the Northern Hemisphere [[34], [35], [36], [37]]. Experimental studies have found that other *Culex* species are also potent vectors for the virus [37]. For example, a German study found that *Cx. torrentium*, which is widely distributed in the Nordic countries, is superior to *Cx. pipiens* in transmitting USUV under experimental conditions [38].

Phylogenetic studies have shown that USUV has been introduced to Europe from Africa several times and has spread further in Europe, and that these circulating virus strains differ from those in Africa [39], which suggests that the virus overwinters in Europe. The genetic variation is indicative of local adaptation of USUV to European populations of *Cx. pipiens* following its introduction. Vector competence, the ability of mosquitoes to become infected and further transmit a virus, has been demonstrated for USUV in *Cx. pipiens* colonies in the Netherlands [36]. In a colony from the United Kingdom, experimental infection with a virus originating in Africa was successful in only one mosquito, indicating limited primary susceptibility, but also showing that infection and dissemination were possible [35]. In Sweden, there is no evidence of circulating autochthonous vector transmission of USUV, but the virus was isolated in 2019 from a dead blackbird found on Öland, an island and province off the southeastern coast of Sweden [40]. This is the northernmost known location of USUV to date. However, an increase in serological evidence and USUV RNA detection in Germany and Poland, suggest that the virus is spreading northward [28,41].

The emergence of West Nile virus (WNV) has highlighted the importance of increased surveillance for mosquito-borne diseases in livestock, mosquitoes, and humans. Cases of USUV may still be under-recognized, and increased surveillance needs to be implemented in many European countries. Identification of potential vectors of USUV or other mosquito-borne viruses in a given geographic area have important implications for outbreak control and early assessment of the risk of virus introduction. Given the known role of *Cx. pipiens* as a vector for WNV and USUV in other European countries and its wide distribution in Sweden, it is important to determine the ability of local *Cx. pipiens* to transmit USUV. In this study, we aimed to evaluate the ability of an Italian strain of USUV to infect Swedish *Cx. pipiens* and assess the potential of these mosquitoes to act as vectors for USUV.

## 2 Methods

### 2.1 Mosquito strain

*Cx. pipiens* biotype *molestus* mosquitoes were originally collected in Gothenburg, Sweden in 2016 and speciated by physiology (autogenous), behavior (non-hibernating, underground breeding) as well as molecular species identification [42]. Mosquitoes were reared at the Zoonosis Science Centre (ZSC) mosquito laboratory, Uppsala University, at 22 °C ± 1 °C at 70% ± 5% relative humidity (RH) under a 16-h light/8-h dark (LD) photoperiod. Adult mosquitoes were continuously provided a 10% sugar solution and once a week offered defibrillated horse blood (Håtuna lab, Uppsala, Sweden) spiked with 5% sugar.

### 2.2 Virus strain and cell lines

The Bologna 2009 USUV strain (GenBank No. HM569263) passage 2 was obtained from Gorben P. Pijlman at the Laboratory of Virology, Wageningen University. This virus was originally isolated from the plasma of a Italian patient who developed neuroinvasive disease following an orthotropic liver transplantation [43]. The Bologna 2009 strain was propagated in C6/36 cells and passage four and five were used in our experiments.

*Aedes albopictus* C6/36 cells (Sigma-Aldrich, Darmstadt, Germany) were grown in Leibovitz's L-15 Medium (L-15) (Gibco™, Thermo Fisher Scientific, Inc., Waltham, MA, USA) supplemented with 10% Fetal Bovine Serum (FBS) (Gibco™, Thermo Fisher Scientific, Inc., Waltham, MA, USA), 10% Tryptose Phosphate Broth (TPB) (Gibco™, Thermo Fisher Scientific, Inc., Waltham, MA, USA) and 100 units per milliliter (U/mL) each of penicillin and streptomycin (penstrep) (Gibco™, Thermo Fisher Scientific, Inc., Waltham, MA, USA) at 28 °C. Infected cells were incubated for 4.5 days in L-15 with 2% FBS, 10% TPB, and 1% penstrep. Viruses were harvested and stored at −80 °C. Viral titer was expressed in plaque forming units per milliliter (PFU/mL). Golden hamster kidney cells clone C13 (BHK21) (Friedrich-Loeffler-Institut, Greifswald, Germany) were grown in Dulbecco's Modified Eagle Medium (DMEM) (Gibco™, Thermo Fisher Scientific, Inc., Waltham, MA, USA) supplemented with 10% FBS and 100 U/mL penstrep at 37 °C with 5% CO_2_.

### 2.3 Virus titration

**Supplementary Fig. 1:**
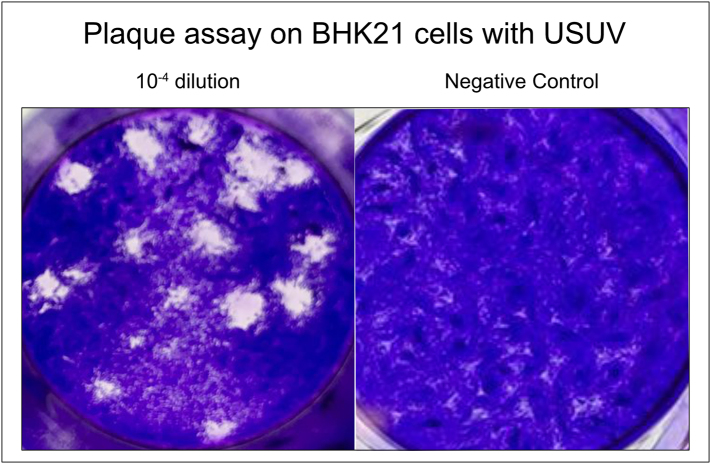
Plaque assay on BHK21 cells with USUV.

Virus titration was performed based on a plaque assay developed for DENV [44] with adjustments for USUV. BHK21 cells were seeded at a density of 1.6*10^5^ cells/well in DMEM supplemented with 5% FBS and 1% penstrep in cell culture treated 24-well plates. Next day a 1% agarose solution (Noble Agar, Thermo Fisher Scientific, Inc., J10907, Waltham, MA, USA) was melted and maintained at 45 °C while preparing MEM (Temin's modification) (2×) (2xMEM) (Gibco™, Thermo Fisher Scientific, Inc., Waltham, MA, USA), supplemented with 14% heat-inactivated FBS and 2% penstrep. This 2xMEM mixture was kept at room temperature. A 10-fold serial dilution of the virus was prepared in DMEM without FBS. The cell monolayers were rinsed using DMEM without FBS before adding 200 μL virus dilution per well. The plate was incubated at 37 °C for 1 h with intermittent shaking every 10 min. Afterward, the supernatant was removed, and an agarose-2xMEM mixture (1:1) was prepared and 600 μL were added to each well. The mixture was allowed to solidify before plates were incubated at 37 °C for three days. On day three, a fixative solution (25% formaldehyde in PBS) was added to each well for 30 min. Subsequently, the agarose was carefully removed, and the cell monolayers were stained with a 1% crystal violet solution (1% crystal violet powder, 20% of 100% ethanol, 25% of 37% formaldehyde in PBS). After a 15-min incubation, the crystal violet solution was removed, and cells were rinsed with water. The viral titer was determined by counting plaques in the first well with fewer than 20 plaques and the titer was calculated as follows: titer = number of plaques × 5 (for 1 mL) × 1/dilution in PFU/mL (Supplementary Fig. 1).

### 2.4 Mosquito infection experiments

Seven to 14 days old female mosquitoes were deprived of sugar solution for 24 h before being offered an infectious blood meal. The infectious blood meal consisted of a 1:5 viral suspension and horse blood. The viral titer of the infectious blood meal was 2 × 10^6^ PFU/mL. Mosquitoes were allowed to feed for 2 to 3 h at 25 °C in the dark on a half cotton pad soaked with the virus-blood mixture. Fully engorged females were transferred at room temperature using a mechanical aspirator to 300 mL plastic cups and maintained at 25 °C ± 1 °C, equal to a warm summer day temperature in Sweden, with a 16-h light/8-h dark cycle for 7, 14, 21, and 28 days representing early, intermediate, late, and very late stages of flavivirus infection in this mosquito species [45]. Mosquitoes were continuously fed a 10% sugar solution. Uninfected control mosquitoes were fed blood without virus and were kept for 21 days.

To investigate the replication of USUV in the body, legs, and saliva of individual mosquitoes, saliva was collected through forced salivation on days 7, 14, 21, and 28 post feeding (PF) [46,47]. In short, female mosquitoes were freeze sedated for 8 min, legs were collected, and the proboscis was inserted into a capillary tube filled with approximately 10 μL of oil for 30 min, after which the oil was collected. The body, legs, and saliva of each mosquito were transferred individually into 1.5 mL microtubes containing 300 mL of mosquito buffer (phosphate-buffered saline (PBS) (Gibco™, Thermo Fisher Scientific, Inc., Waltham, MA, USA) supplemented with 20% FBS, 1% penstrep, and 1% amphotericin B (Gibco™, Thermo Fisher Scientific, Inc., Waltham, MA, USA). The body and leg samples were then homogenized using 5 mm stainless steel beads and the TissueLyser II (Qiagen, Hilden, Germany) bead mill set to a frequency of 25 Hz for 2 min.

For reliable interpretation of USUV detection in samples from early timepoints, 12 mosquitoes were also offered heat-inactivated USUV. Viral inactivation was accomplished through incubation of the viral stock at 56 °C for 30 min as previously described effective for inactivating other Flavi and related viruses [[48], [49], [50]]. The mosquitoes were provided a blood meal with heat-inactivated USUV at the same concentration as in the experiments with infectious virus, and were kept under the same conditions for seven days. After incubation the whole mosquitoes were used for RNA extraction and RTqPCR (see below) to control for detection of residual, undigested viral components originating from the virus-containing blood meal. The heat-inactivated virus was also analyzed by both plaque assay and RTqPCR to control for inactivation and potential loss of RNA.

### 2.5 USUV detection in mosquito bodies, legs, and saliva by real-time RT-PCR

Bodies, legs and saliva of all blood-fed mosquitoes that survived the incubation were analyzed. Viral RNA was extracted using the QIAamp^Ⓡ^ Viral RNA Mini Kit (Qiagen, Hilden, Germany) according to manufacturer's instructions. Elution was carried out in water and samples were stored short time in −20 °C until RT-qPCR analysis and thereafter transferred to −80 °C for long term storage. USUV was detected in a RT-qPCR assay using the QuantiTect Probe RT-PCR kit (Qiagen, Hilden, Germany) according to manufacturer's instructions. The forward primer 3′-caaagctggacagacatcccttac-5′, reverse primer 3′-cgtagatgttttcagcccacgt-5′ and probe 6FAM-aagacatatggtgtggaagcctgataggca-TMR were previously published and amplify a 103 base pair (bp) sequence in the NS5 gene [51]. The PCR reaction mix contained 12.5 μL 2× QuantiTect Probe RT-PCR Master Mix (HotStarTaq® DNA Polymerase, QuantiTect Probe RT-PCR Buffer, dNTP mix, including dUTP, ROX™ passive reference dye, and 8 mM MgCl2), 6 μL RNAse-free water, 0.5 μL forward primer, 0.5 μL reverse primer, 0.25 μL probe, and 0.25 μL QuantiTect RT Mix (Omniscript® Reverse Transcriptase and Sensiscript® Reverse Transcriptase) per reaction. A total of 20 μL master mix was used for each reaction well and 5 μL of extracted RNA was used as template. All PCRs were run using the CFX Connect Real-Time PCR Detection System (Bio-Rad, Hercules, California, USA) with 30 min reverse transcription (RT) at 50 °C, 15 min initial activation at 95 °C, followed by 40 cycles of 15 s at 94 °C and 1 min at 60 °C. Samples were considered positive when a FAM signal was obtained (≤40 Ct) and, for Ct values between 30 and 40, amplification was confirmed by visualization of a correctly sized amplification product on an 1,5% agarose gel. All samples were at least run twice by the RT-qPCR, once on the gel and only counted as positive if both PCR replicates yielded positive results.

### 2.6 Data analysis

Mosquito bodies were analyzed to determine the infection rate (IR), calculated as the number of USUV-positive bodies divided by the total number of surviving blood fed females per timepoint. Dissemination rates (DR) of the virus into the legs was calculated as the number of USUV-positive legs divided by the number of USUV-positive bodies. Detection of the virus in the saliva was analyzed to determine the transmission rate (TR), calculated as the number of USUV-positive saliva samples divided by the number of USUV-positive bodies. Transmission efficiency (TE) was defined as the total number of mosquitoes where all three collected sample types were USUV-positive out of the total number of mosquitoes analyzed.

Chi-square and simple logistic regression tests were performed using GraphPad Prism version 10.0.0 for MacOS, GraphPad Software, Boston, Massachusetts USA, www.graphpad.com, to test for significant differences (**** = p *<* 0.0001, *** = p *<* 0.001, ** = p *<* 0.01, * = p *<* *0.05, ns = not significant*) in infection, dissemination, and transmission between timepoints.

## 3 Results

### 3.1 Feeding and survival rates of mosquitoes at laboratory conditions

Feeding rates ranged from 72% to 98%, and survival rates dropped from 95% at 7 days PF to 78% at 14 days PF (Table 1). Survival rates dropped further to 61% and 63% at 21 and 28 days PF, respectively. Feeding rates for control mosquitoes fed with blood without virus were 90% and the survival rate at 21 days was 97%. The number of mosquitoes sampled at each time point ranged from 42 to 66 individuals (Table 1).

**Table 1:** Mosquito feeding and survival rates for each timepoint.

Days PF	Total mosquitoes	Feeding rate	Survival rate
		No. fed females (percentage of total)	No. at collection (percentage of fed)
Offered blood meal containing 2*10^6^ PFU/mL USUV			
7	61	44 (72%)	42 (95%)
14	77	67 (87%)	52 (78%)
21	121	87 (79%)	58 (61%)
28	107	105 (98%)	66 (63%)

Offered blood meal without virus			
21	40	36 (90%)	35 (97%)

### 3.2 Oral susceptibility, dissemination and transmission rates of USUV

**Fig. 1:**
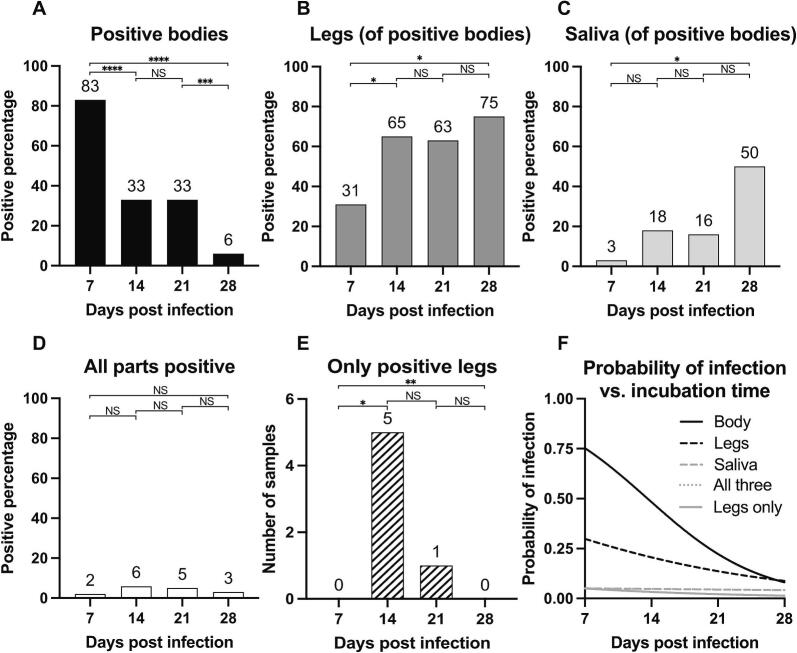
USUV positive body, leg, and saliva samples in Cx. pipiens females after ingestion of virus and subsequent incubation for 7 to 28 days and the calculated probability of infection compared to incubation time. Mosquitoes were fed a blood-meal containing 2 × 10^6^ PFU/mL USUV. Virus was detected in bodies (A), bodies and legs (B), and bodies, legs and saliva (C) at 7 (*n* = 42), 14 (*n* = 52), 21 (*n* = 58), and 28 (*n* = 66) days post feeding by RT-qPCR. (A) shows percentages of infected compared to non-infected after ingestion of a USUV spiked blood meal. (B) shows percentages of infected mosquitoes with USUV positive legs (C) shows percentages of infected mosquitoes with positive saliva (D) shows the percentage of mosquitoes in which virus was detected in all three samples taken from the same individual compared to all mosquitoes ingesting a USUV spiked blood meal. (E) shows the number of samples in which virus was detected in the leg samples but in no other part pf the mosquito compared to all tested mosquitoes. Chi-square-test was used for statistical comparison in (A)-(E); **** = *p* < 0.0001, *** = *p* < 0.001, ** = *p* < 0.01, * = *p* < 0.05, ns = not significant. Simple logistic regression was performed to understand the probability of infection over time. Probability of infection versus incubation time is shown in (F), significant lines are in black, not significant in grey. The lines for saliva and all three parts positive are overlapping.

The IR, DR, and TR were calculated for *Cx. pipiens* females at 7, 14, 21, and 28 days PF. USUV was detected in the bodies, legs, and saliva of mosquitoes at all time points (Table 2, Supplementary Table 1). The numbers of infected samples for each timepoint and specimen are found in Table 2. Percentages given are percentages of tested for the IR, and percentage of positive bodies for DR and TR. There were significantly more USUV positive bodies at 7 days PF (83%) than at 14 and 21 days PF (both 33%) (*p* < 0.0001 [*χ*^2^ = 24.11, df = 1]) (Fig. 1A). It decreased significantly to 6% at day 28 PF (*p* < 0.001 [*χ*^2^ = 14.56, df = 1]) (Fig. 1A).

The DR, i.e., the number of mosquitoes with a USUV-positive body in which USUV was also detected in the legs, was significantly higher at 14 and 21 days PF (64% and 63%, respectively) than at 7 days PF (31%) (*p* = 0.023 [*χ*^2^ = 5.19, df = 1]) (Fig. 1B). The DR increased further from 21 to 28 days PF (75%) but not significantly (*p* = 0.651 [*χ*^2^ = 0.20, df = 1]) (Table 2, Fig. 1B).

**Table 2:** Infection, dissemination, and transmission rates of USUV in Cx. pipiens mosquitoes at four timepoints.

Days PF	No. tested	Infection rate (IR) (percentage of tested)	Dissemination rate (DR) (percentage of infected)	Transmission rate (TR) (percentage of infected)
7	42	35 (83%)	11 (31%)	1 (3%)
14	52	17 (33%)	11 [5] (65%)	3 (18%)
21	58	19 (33%)	12 [1] (63%)	3 (16%)
28	66	4 (6%)	3 (75%)	2 (50%)

Note: At 14 and 21 days PF positive leg samples with corresponding negative body and saliva samples are added in [] and not counted in the percentage calculations.

Virus in saliva was detected already at timepoint 7 and the TR increased slightly over the four timepoints peaking at 50% at 28 days PF (*p* = 0.029 [*χ*^2^ = 9.012, df = 3]) (Fig. 1C). None of the calculated rates (IR, DR, and TR) showed any significant differences at 14 days compared with 21 days PF (*p* = 0.994 [*χ*^2^ = 5.48*10^−5^, df = 1]; *p* = 0.923 [*χ*^2^ = 0.009 df = 1]; *p* = 0.022 [*χ*^2^ = 0.881, df = 1]). There was no significant difference of TE, i.e. the percentage of mosquito individuals containing USUV in all three body parts, observed over the four timepoints (*p* = 0.568 [*χ*^2^ = 2.02, df = 3]) (Fig. 1D).

Logistic regression was used to analyze the relationship between the incubation time and whether mosquitoes were infected or not (Fig. 1F). It was found, keeping all other variables constant, that the probability of infection of the mosquito body decreased significantly by −15.52% (95% CI [−11.53%, −19.73%], *p* < 0.0001) per day PF. Also, the probability of infection in the legs decreased significantly by −6.83% (95% CI [−2.35%, −11.31%], *p* = 0.004) per day PF. It was found that the slight decrease in probability of infection in the saliva, −0.97% (95% CI [7.8%, −8.84%], *p* = 0.816), and for all three samples to be infected, −0.97% (95% CI [7.8%, −8.84%], p = 0.816) was not significant over time.

The plaque-assay performed after heat-inactivation of virus confirmed the absence of viable viral particles, and RT-qPCR revealed only marginal RNA degradation following heat treatment. USUV RNA could not be detected in the body, legs, and saliva at 7 days PF in mosquitoes fed with heat-inactivated virus, providing evidence that the USUV detected in mosquitoes at the 7-day timepoint were a result of active viral replication within the mosquito body, rather than residual components from the initial virus-containing blood meal.

At 14 and 21 days PF, five leg samples and one leg sample, respectively, tested positive for USUV, despite that the corresponding body and saliva samples yielding negative results (Fig. 1E). The confirmation of these samples through repeated analyses employing both PCR and gel electrophoresis substantiated their authenticity and ruled out potential methodological discrepancies. The Ct values were below the cutoff value of 40 (at 14 days 27.33–32,13, at 21 days 27.64) and showed correct size amplicons in the gel analyses. No instances of such samples were identified at either the 7 or 28 days PF. The probability of detecting only positive legs decreased over time by −6.05% (95% CI [4.5%, −16.54%], *p* = 0.259), but not significantly.

## 4 Discussion

In this study, the susceptibility and transmission potential of *Cx. pipiens* mosquitoes from Sweden were investigated for the first time using the USUV Bologna 2009 strain. According to our findings, Swedish *Cx. pipiens* mosquitoes are susceptible to USUV infection and can potentially transmit USUV between hosts, as the virus can be detected in the saliva of infected mosquitoes. USUV is an emerging arbovirus that has spread beyond its historical range into new regions [52]. Studying the vector competence of Swedish mosquitoes allows us to monitor a potential spread of the virus into new areas. This is particularly important given the changing climate and increasing global travel and trade patterns that may facilitate the introduction of new pathogens. Understanding the role of different mosquito species in USUV transmission can provide insights into the ecological dynamics of the virus. As of Summer 2023, there have not been any reports of USUV detected in mosquitoes in Sweden, no reports of human cases, and no evidence of USUV circulation in the bird population, with the exception of a single USUV isolate from a blackbird sampled on Öland, a Swedish island in the Baltic Sea, in 2019 [40,53]. Since 2019, The Swedish National Veterinary Institute (SVA) conducts investigations of deceased Swedish birds to assess the presence of USUV and WNV within the country [54].

Although the IR, DR, and TR (Fig. 1A-C) vary between different time points in our experiments, the overall transmission efficiency, meaning the percentage of mosquitoes that have USUV in body as well as legs and saliva, and therefore are likely to be able to transmit USUV, is consistent across all observed time points (Fig. 1D). The IR decreases over time and the DR increases slightly. In mosquitoes, viruses face multiple physical barriers during infection, and it is conclusive that the rates of dissemination and transmission in mosquitoes with positive bodies increase over time (Fig. 1B, C) [55]. The drop in IR observed over time is less expected, but not unique to this study [[56], [57], [58], [59], [60]]. It has been suggested that observations of decreasing IRs could be due to mortality in infected mosquitoes or that mosquitoes could clear the virus at later time points [60]. However, neither virus induced mortality or viral clearance have strong support in the literature and thus further studies are needed to explain IR decreasing with time [61].

The consistent percentage of mosquitoes testing positive for the virus in body, leg and saliva over time (Fig. 1D) presents an interesting finding in our study. The results indicate that a proportion, ranging from 2% to 6%, of mosquitoes harbor virus in their gut, hemocoel, and saliva as early as 7 days post an infectious blood meal, and maintain the infection for at least the tested 28 days. After feeding mosquitoes heat-inactivated USUV, no viral RNA could be detected after 7 days, thus we conclude that detection of USUV RNA in bodies, legs and saliva 7 days post feeding on infectious blood is due to virus replication in the mosquito. This is in agreement with previous studies on blood-meal digestion times, reporting that digestion usually takes less than five days at temperatures of 25 °C or higher [[62], [63], [64]].

The observed pattern is further supported by the non-significant difference in the probability of all three sample-types from a single mosquito showing infection over time (Fig. 1F). This suggests that the extrinsic incubation period (EIP), i.e., the duration between infection and the presence of the virus in saliva, may be relatively short. Given the limited available information on the EIP of USUV in *Culex*, insights from WNV studies can be utilized for comparison. Notably, transmission of different WNV strains in *Cx. pipiens* and *Cx. quinquefasciatus* can occur as early as five days after feeding on a viral titer of 1 × 10^8^ PFU/mL and incubation at 27 °C [65]. Another study of *Cx. pipiens* revealed transmission of WNV to suckling mice as early as eight days using 2.7 × 10^6^ PFU/mL at 26 °C [66]. Furthermore, the presence of WNV in the saliva of *Cx. pipiens* was detected as soon as 3 days post-infection, peaking on day 14 after ingesting a viral titer of 1.6 × 10^8^ PFU/mL and incubation at 28 °C [67]. Such a short incubation period is of particular significance as mosquito mortality rates can be high in natural settings, especially at high temperatures. A reduced incubation period increases the likelihood that infected mosquitoes will survive long enough to continue transmitting the virus to new hosts [55].

Our experiments investigated the vector competence of Swedish *Cx. pipiens* mosquitoes for the USUV strain Bologna `09 at 2*10^6^ PFU/mL. The same strain was used in Fros et al.'s comparable study from the Netherlands [36]. Fros et al.'s study on Dutch mosquitoes investigated vector competence at 18 °C, 23 °C and 28 °C with 4*10^7^ TCID50/mL virus in blood. Our results showed a lower infection rate of 33% at 14 days PF, with a saliva positivity of 6% at 25 °C when compared to Fros et al.´s reported infection rate of 53% at 23 °C. The infection rate was 80% and the proportion of mosquitoes with positive saliva was 69% after 14 days PF at 28 °C. They also showed, that the infection rate further dropped to 11% at 18 °C. In another study, Holicki et al. infected German and Serbian *Cx. pipiens molestus* mosquitoes at 25 °C with 10^7.4^ TCID50/mL of a USUV Africa 2 strain (KU664608) [38]. Both populations exhibited higher infection rates (over 80%) in later stages of infection. Serbian mosquitoes showed 100% dissemination rates, while the dissemination rates of the German mosquitoes ranged from 37.5% to 100% at 14 days PF, slightly higher than our Swedish results [38]. Interestingly, the proportion of mosquitoes with positive saliva was notably higher in both German (16% to 100%) and Serbian (up to 40%) mosquitoes [38]. They also showed that a lower titer of virus (10^5.1^ TCID50/mL) decreased the infection rates drastically to 5.6% and no dissemination or transmission was observed. The transmission efficiency, meaning the proportion of mosquitoes that were positive in body, legs and wings, and saliva was slightly higher (30% and 12.5%) than what we found at 14 days PF (6%). However, their smaller sample size (10 and 16 German and 26 Serbian mosquitoes) compared to our study (42 to 66 mosquitoes per timepoint) could also influence the results.

The presence of positive leg samples at 14 days PF and 21 days PF (five and one respectively, ct 28.2 ± 1.8), despite bodies testing negative for USUV in the same individuals, is noteworthy. These samples repeatedly produced RT-qPCR signals and amplification bands on the gel, thus we cannot explain them with any methodological error. In the work of Holicki et al., they discovered positive saliva samples in mosquitoes that were negative in the leg and wing samples analyzed, although they did not delve into further discussion, considering these findings as errors [38]. Many studies do not analyze the legs or saliva of mosquitoes with negative bodies, and therefore little is known about this finding from other studies. Fros et al. discovered an intriguing increase in RNA interference activity (RNAi) in mosquitoes infected with USUV [36]. RNAi serves as the primary antiviral response in mosquitoes and other insects [68]. If the mosquito was initially infected with the virus and has established disseminated infection with virus circulating in the hemocoel, it may be possible that an RNAi response has initiated the clearance of the virus from the gut and most of the hemocoel, but not yet completely in the more distant legs, leading to isolated detection of virus in the legs. More studies analyzing all samples from all mosquitoes are needed to verify this hypothesis as a phenomenon. Additionally immune responses of mosquitoes against viral infections need to be studied in greater depth to understand and evaluate the possibility of clearance of an infection over time.

## 5 Conclusions

This study demonstrated that Swedish *Cx. pipiens molestus* mosquitoes are vector competent for USUV. Transmission rates were <20% up to 21 days PF and reached 50% after 28 days, raising the question of what risk USUV poses to Sweden. This risk is influenced by mosquito longevity and field infection rates in the *Culex* population, thus studying all these factors is crucial to prevent future outbreaks of USUV disease.

The following are the supplementary data related to this article.

Supplementary Table 1 Summary of PCR results for all analyzed samples. Supplementary Table 1

## Author contributions

Conceptualization, J.K., J.C.H., and Å.L.; Data curation, J.K.; Formal analysis, J.K., E.B.F., and P.H.; Funding acquisition, Å.L. and J.C.H.; Investigation, J.K., E.B.F., P.H., Å.L., Methodology, J.K. and J.C.H.; Project administration, J.K. and J.C.H.; Supervision, J.K. and J.C.H.; Visualization, J.K; Writing - original draft J.K.; and Writing - review & editing, all authors. All authors have read and agreed to the published version of the manuscript.

## Funding

This research was funded by the European Union's Horizon 2020 research innovation program (Grant no. 874735 (VEO)), and the SciLifeLab Pandemic Preparedness projects (LPP1–007 and REPLP1:005).

## CRediT authorship contribution statement

**Janina Krambrich:** Conceptualization, Data curation, Formal analysis, Investigation, Methodology, Project administration, Supervision, Validation, Visualization, Writing – original draft, Writing – review & editing. **Emma Bole-Feysot:** Formal analysis, Investigation, Writing – review & editing. **Patrick Höller:** Formal analysis, Writing – review & editing. **Åke Lundkvist:** Funding acquisition, Writing – review & editing. **Jenny C. Hesson:** Funding acquisition, Writing – review & editing.

## Declaration of competing interest

The authors declare that they have no known competing financial interests or personal relationships that could have appeared to influence the work reported in this paper.

## Data Availability

The authors confirm that the data supporting the findings of this study are available within the atricle and its supplementary materials.

## References

[1] Rathore A.P.S., St John A.L. (2020). Cross-reactive immunity among flaviviruses. Front. Immunol..

[2] Lindenbach B., Rice C. (2001).

[3] Calzolari M., Bonilauri P., Bellini R., Albieri A., Defilippo F., Maioli G., Galletti G., Gelati A., Barbieri I., Tamba M., Lelli D., Carra E., Cordioli P., Angelini P., Dottori M. (2010). Evidence of simultaneous circulation of West Nile and Usutu viruses in mosquitoes sampled in Emilia-Romagna Region (Italy) in 2009. PLoS One.

[4] Calzolari M., Gaibani P., Bellini R., Defilippo F., Pierro A., Albieri A., Maioli G., Luppi A., Rossini G., Balzani A., Tamba M., Galletti G., Gelati A., Carrieri M., Poglayen G., Cavrini F., Natalini S., Dottori M., Sambri V., Angelini P., Bonilauri P. (2012). Mosquito, bird and human surveillance of West Nile and Usutu viruses in Emilia-Romagna Region (Italy) in 2010. PLoS One.

[5] Cadar D., Simonin Y. (2022). Human Usutu virus infections in Europe: a new risk on horizon?. Viruses.

[6] Pacenti M., Sinigaglia A., Martello T., De Rui M.E., Franchin E., Pagni S., Peta E., Riccetti S., Milani A., Montarsi F., Capelli G., Doroldi C.G., Bigolin F., Santelli L., Nardetto L., Zoccarato M., Barzon L. (2019). Clinical and virological findings in patients with Usutu virus infection, northern Italy, 2018. Euro Surveill..

[7] Medrouh B., Lafri I., Beck C., Leulmi H., Akkou M., Abbad L., Lafri M., Bitam I., Lecollinet S. (2020). First serological evidence of West Nile virus infection in wild birds in northern Algeria. Comp. Immunol. Microbiol. Infect. Dis..

[8] Sejvar J.J. (2014). Clinical manifestations and outcomes of West Nile virus infection. Viruses.

[9] Nikolay B., Diallo M., Boye C.S., Sall A.A. (2011). Usutu virus in Africa. Vector Borne Zoonotic Dis..

[10] Tinto B., Kaboré D.P.A., Kagoné T.S., Constant O., Barthelemy J., Kiba-Koumaré A., Van de Perre P., Dabiré R.K., Baldet T., Gutierrez S. (2022). Screening of circulation of Usutu and West Nile Viruses: A one health approach in humans, domestic animals and mosquitoes in Burkina Faso, West Africa. Microorganisms.

[11] Raulino R., Thaurignac G., Butel C., Villabona-Arenas C.J., Foe T., Loul S., Ndimbo-Kumugo S.-P., Mbala-Kingebeni P., Makiala-Mandanda S., Ahuka-Mundeke S. (2021). Multiplex detection of antibodies to chikungunya, O’nyong-nyong, Zika, dengue, West Nile and Usutu viruses in diverse non-human primate species from Cameroon and the Democratic Republic of Congo. PLoS Negl. Trop. Dis..

[12] Williams M.C., Simpson D.I., Haddow A.J., Knight E.M. (1964). The isolation of West Nile virus from man and of Usutu virus from the bird-biting mosquito Mansonia Aurites (Theobald) in the Entebbe area of Uganda. Ann. Trop. Med. Parasitol..

[13] Vázquez A., Jiménez-Clavero M.A., Franco L., Donoso-Mantke O., Sambri V., Niedrig M., Zeller H., Tenorio A. (2011). Usutu virus – potential risk of human disease in Europe. Eurosurveillance.

[14] Clé M., Beck C., Salinas S., Lecollinet S., Gutierrez S., Van de Perre P., Baldet T., Foulongne V., Simonin Y. (2019). Usutu virus: a new threat?. Epidemiol. Infect..

[15] Durand B., Haskouri H., Lowenski S., Vachiery N., Beck C., Lecollinet S. (2016). Seroprevalence of West Nile and Usutu viruses in military working horses and dogs, Morocco, 2012: dog as an alternative WNV sentinel species?. Epidemiol. Infect..

[16] Ben Hassine T., De Massis F., Calistri P., Savini G., BelHaj Mohamed B., Ranen A., Di Gennaro A., Sghaier S., Hammami S. (2014). First detection of co-circulation of West Nile and Usutu viruses in equids in the south-west of Tunisia. Transbound. Emerg. Dis..

[17] Diagne M.M., Ndione M.H.D., Di Paola N., Fall G., Bedekelabou A.P., Sembène P.M., Faye O., Zanotto P.M.A., Sall A.A. (2019). Usutu virus isolated from rodents in senegal. Viruses.

[18] Mani P., Rossi G., Perrucci S. (1998). Mortality of Turdus merula in Tuscany. Selezione Veterinaria.

[19] Weissenböck H., Bakonyi T., Rossi G., Mani P., Nowotny N. (2013). Usutu virus, Italy, 1996. Emerg. Infect. Dis..

[20] Weissenböck H., Kolodziejek J., Fragner K., Kuhn R., Pfeffer M., Nowotny N. (2003). Usutu virus activity in Austria, 2001–2002. Microbes Infect..

[21] Hubálek Z., Rudolf I., Čapek M., Bakonyi T., Betášová L., Nowotny N. (2014). Usutu virus in blackbirds (Turdus merula), Czech Republic, 2011–2012. Transbound. Emerg. Dis..

[22] Jöst H., Bialonski A., Maus D., Sambri V., Eiden M., Groschup M.H., Günther S., Becker N., Schmidt-Chanasit J. (2011). Isolation of usutu virus in Germany. Am. J. Trop. Med. Hyg..

[23] Becker N., Jöst H., Ziegler U., Eiden M., Höper D., Emmerich P., Fichet-Calvet E., Ehichioya D.U., Czajka C., Gabriel M., Hoffmann B., Beer M., Tenner-Racz K., Racz P., Günther S., Wink M., Bosch S., Konrad A., Pfeffer M., Groschup M.H., Schmidt-Chanasit J. (2012). Epizootic emergence of Usutu virus in wild and captive birds in Germany. PLoS One.

[24] Bakonyi T., Erdélyi K., Ursu K., Ferenczi E., Csörgő T., Lussy H., Chvala S., Bukovsky C., Meister T., Weissenböck H., Nowotny N. (2007). Emergence of Usutu virus in Hungary. J. Clin. Microbiol..

[25] Vázquez A., Ruiz S., Herrero L., Moreno J., Molero F., Magallanes A., Sánchez-Seco M.P., Figuerola J., Tenorio A. (2011). West Nile and Usutu viruses in mosquitoes in Spain, 2008-2009. Am. J. Trop. Med. Hyg..

[26] Steinmetz H.W., Bakonyi T., Weissenböck H., Hatt J.-M., Eulenberger U., Robert N., Hoop R., Nowotny N. (2011). Emergence and establishment of Usutu virus infection in wild and captive avian species in and around Zurich, Switzerland—genomic and pathologic comparison to other central European outbreaks. Vet. Microbiol..

[27] Folly A.J., Lawson B., Lean F.Z., McCracken F., Spiro S., John S.K., Heaver J.P., Seilern-Moy K., Masters N., Hernández-Triana L.M., Phipps L.P., Nuñez A., Fooks A.R., Cunningham A.A., Johnson N., McElhinney L.M. (2020). Detection of Usutu virus infection in wild birds in the United Kingdom, 2020. Eurosurveillance.

[28] Bażanów B., Jansen van Vuren P., Szymański P., Stygar D., Frącka A., Twardoń J., Kozdrowski R., Pawęska J.T. (2018). A survey on West Nile and Usutu viruses in horses and birds in Poland. Viruses.

[29] Barbic L., Vilibic-Cavlek T., Listes E., Stevanovic V., Gjenero-Margan I., Ljubin-Sternak S., Pem-Novosel I., Listes I., Mlinaric-Galinovic G., Di Gennaro A., Savini G. (2013). Demonstration of Usutu virus antibodies in horses, Croatia. Vector-Borne Zoonot. Dis..

[30] Klobucar A., Savic V., Curman Posavec M., Petrinic S., Kuhar U., Toplak I., Madic J., Vilibic-Cavlek T. (2021). Screening of mosquitoes for West Nile virus and Usutu virus in Croatia, 2015–2020. Trop. Med. Infect. Dis..

[31] Rijks J.M., Kik M.L., Slaterus R., Foppen R., Stroo A., Ijzer J., Stahl J., Gröne A., Koopmans M., van der Jeugd H.P., Reusken C. (2016). Widespread Usutu virus outbreak in birds in the Netherlands, 2016. Euro Surveill..

[32] Mancuso E., Toma L., Pascucci I., d’Alessio S.G., Marini V., Quaglia M., Riello S., Ferri A., Spina F., Serra L., Goffredo M., Monaco F. (2022). Direct and indirect role of migratory birds in spreading CCHFV and WNV: a multidisciplinary study on three stop-over islands in Italy. Pathogens.

[33] Mancuso E., Cecere J.G., Iapaolo F., Di Gennaro A., Sacchi M., Savini G., Spina F., Monaco F. (2022). West Nile and Usutu virus introduction via migratory birds: a retrospective analysis in Italy. Viruses.

[34] Engler O., Savini G., Papa A., Figuerola J., Groschup M.H., Kampen H., Medlock J., Vaux A., Wilson A.J., Werner D. (2013). European surveillance for West Nile virus in mosquito populations. Int. J. Environ. Res. Public Health.

[35] Hernández-Triana L.M., de Marco M.F., Mansfield K.L., Thorne L., Lumley S., Marston D., Fooks A.A., Johnson N. (2018). Assessment of vector competence of UK mosquitoes for Usutu virus of African origin. Parasit. Vectors.

[36] Fros J.J., Miesen P., Vogels C.B., Gaibani P., Sambri V., Martina B.E., Koenraadt C.J., van Rij R.P., Vlak J.M., Takken W., Pijlman G.P. (2015). Comparative Usutu and West Nile virus transmission potential by local Culex pipiens mosquitoes in North-Western Europe. One Health.

[37] Tolsá-García M.J., Wehmeyer M.L., Lühken R., Roiz D. (2023). Worldwide transmission and infection risk of mosquito vectors of West Nile, St. Louis encephalitis, Usutu and Japanese encephalitis viruses: a systematic review. Sci. Rep..

[38] Holicki C.M., Scheuch D.E., Ziegler U., Lettow J., Kampen H., Werner D., Groschup M.H. (2020). German Culex pipiens biotype molestus and Culex torrentium are vector-competent for Usutu virus. Parasit. Vectors.

[39] Siljic M., Sehovic R., Jankovic M., Stamenkovic G., Loncar A., Todorovic M., Stanojevic M., Cirkovic V. (2023). Evolutionary dynamics of Usutu virus: worldwide dispersal patterns and transmission dynamics in Europe. Front. Microbiol..

[40] Neimane A., Propst M. (2019). Statens Veterinärmedicinska Anstalt.

[41] Vilibic-Cavlek T., Petrovic T., Savic V., Barbic L., Tabain I., Stevanovic V., Klobucar A., Mrzljak A., Ilic M., Bogdanic M., Benvin I., Santini M., Capak K., Monaco F., Listes E., Savini G. (2020). Epidemiology of Usutu virus: The European scenario. Pathogens.

[42] Hesson J.C., Schäfer M., Lundström J.O. (2016). First report on human-biting Culex pipiens in Sweden. Parasit. Vectors.

[43] Cavrini F., Gaibani P., Longo G., Pierro A.M., Rossini G., Bonilauri P., Gerunda G.E., Di Benedetto F., Pasetto A., Girardis M., Dottori M., Landini M.P., Sambri V. (2009). Usutu virus infection in a patient who underwent orthotropic liver transplantation, Italy, August-September 2009. Eurosurveillance.

[44] Monteil V.M. (2013).

[45] Anderson S.L., Richards S.L., Tabachnick W.J., Smartt C.T. (2010). Effects of West Nile virus dose and extrinsic incubation temperature on temporal progression of vector competence in Culex pipiens quinquefasciatus. J. Am. Mosq. Control Assoc..

[46] Heitmann A., Jansen S., Lühken R., Leggewie M., Schmidt-Chanasit J., Tannich E. (2018). Forced salivation as a method to analyze vector competence of mosquitoes. J. Vis. Exp..

[47] Dubrulle M., Mousson L., Moutailler S., Vazeille M., Failloux A.-B. (2009). Chikungunya virus and Aedes mosquitoes: saliva is infectious as soon as two days after oral infection. PLoS One.

[48] Madani T.A., Abuelzein E.-T.M.E., Azhar E.I., Al-Bar H.M.S. (2014). Thermal inactivation of Alkhumra hemorrhagic fever virus. Arch. Virol..

[49] Idris F., Muharram S.H., Zaini Z., Diah S. (2018). Effectiveness of physical inactivation methods of dengue virus: heat-versus UV-inactivation. bioRxiv.

[50] Fang Y., Brault A.C., Reisen W.K. (2009). Comparative thermostability of West Nile, St. Louis encephalitis, and western equine encephalomyelitis viruses during heat inactivation for serologic diagnostics. Am. J. Trop. Med. Hyg..

[51] Nikolay B., Weidmann M., Dupressoir A., Faye O., Boye C.S., Diallo M., Sall A.A. (2014). Development of a Usutu virus specific real-time reverse transcription PCR assay based on sequenced strains from Africa and Europe. J. Virol. Methods.

[52] Roesch F., Fajardo A., Moratorio G., Vignuzzi M. (2019). Usutu virus: an arbovirus on the rise. Viruses.

[53] Hesson J.C., Lundin E., Lundkvist Å., Lundström J.O. (2019). Surveillance of mosquito vectors in southern Sweden for Flaviviruses and Sindbis virus. Infect. Ecol. Epidemiol..

[54] (2023). Statens Veterinärmedicinska Anstalt Övervakning av West Nile-Virus Och Usutu-Virus. https://www.sva.se/amnesomraden/smittlage/overvakning-av-west-nile-virus-och-usutu-virus/.

[55] Weaver S.C. (2020). Incrimination of mosquito vectors. Nat. Microbiol..

[56] Zhu C., Jiang Y., Zhang Q., Gao J., Li C., Li C., Dong Y., Xing D., Zhang H., Zhao T., Guo X., Zhao T. (2021). Vector competence of Aedes aegypti and screening for differentially expressed microRNAs exposed to Zika virus. Parasit. Vectors.

[57] Azar S.R., Weaver S.C. (2020). Vector competence analyses on Aedes aegypti mosquitoes using Zika virus. J. Vis. Exp..

[58] Limoh B.K., Tchouassi D.P., Chepkorir E., Musimbi B., Ongus J., Sang R. (2023). Vector competence of a coastal population of <i>Aedes aegypti</i> for dengue 2 and 3 virus serotypes in Kenya. Biomed. Res. Int..

[59] Martinet J.-P., Bohers C., Vazeille M., Ferté H., Mousson L., Mathieu B., Depaquit J., Failloux A.-B. (2023). Assessing vector competence of mosquitoes from northeastern France to West Nile virus and Usutu virus. PLoS Negl. Trop. Dis..

[60] Chapman G.E., Sherlock K., Hesson J.C., Blagrove M.S.C., Lycett G.J., Archer D., Solomon T., Baylis M. (2020). Laboratory transmission potential of British mosquitoes for equine arboviruses. Parasit. Vectors.

[61] Cheng G., Liu Y., Wang P., Xiao X. (2016). Mosquito defense strategies against viral infection. Trends Parasitol..

[62] Al-Rashidi H.S., Alghamdi K.M., Al-Otaibi W.M., Al-Solami H.M., Mahyoub J.A. (2022). Effects of blood meal sources on the biological characteristics of Aedes aegypti and Culex pipiens (Diptera: Culicidae). Saudi J. Biol. Sci..

[63] Gooding R.H. (1966). Physiological aspects of digestion of the blood meal by Aedes aegypti (Linnaeus) and Culex fatigans Wiedemann. J. Med. Entomol..

[64] Gray E.M., Bradley T.J. (2003). Metabolic rate in female *Culex tarsalis* (Diptera: Culicidae) : age, size, activity, and feeding effects. J. Med. Entomol..

[65] Romo H., Papa A., Kading R., Clark R., Delorey M., Brault A.C. (2018). Comparative vector competence of north American Culex pipiens and Culex quinquefasciatus for African and European lineage 2 West Nile viruses. Am. J. Trop. Med. Hyg..

[66] Anderson J.F., Main A.J., Delroux K., Fikrig E. (2014). Extrinsic incubation periods for horizontal and vertical transmission of West Nile virus by Culex pipiens pipiens (Diptera: Culicidae). J. Med. Entomol..

[67] Zakhia R., Mousson L., Vazeille M., Haddad N., Failloux A.-B. (2018). Experimental transmission of West Nile virus and Rift Valley fever virus by Culex pipiens from Lebanon. PLoS Negl. Trop. Dis..

[68] Blair C.D. (2011). Mosquito RNAi is the major innate immune pathway controlling arbovirus infection and transmission. Future Microbiol..

