# Supplementary material for: Vector competence of Swedish Culex pipiens mosquitoes for Usutu virus
Source: One Health. 2024 Mar 9;18:100707. doi: 10.1016/j.onehlt.2024.100707 (PMC10945277; doi:10.1016/j.onehlt.2024.100707)
Supplement: Supplementary Table 1 — Summary of PCR results for all analyzed samples. [file mmc2.docx]

Supplementary Table 1: Summary of PCR results for all analyzed samples

| **Mosquito** | **Day** | **Body** | **Legs** | **Saliva** | **Mosquito** | **Day** | **Body** | **Legs** | **Saliva** | **Mosquito** | **Day** | **Body** | **Legs** | **Saliva** | **Mosquito** | **Day** | **Body** | **Legs** | **Saliva** |
| --- | --- | --- | --- | --- | --- | --- | --- | --- | --- | --- | --- | --- | --- | --- | --- | --- | --- | --- | --- |
| 1 | 7 | + | - | - | 56 | 14 | - | - | - | 111 | 21 | + | + | - | 166 | 28 | - | - | - |
| 2 | 7 | - | - | - | 57 | 14 | - | - | - | 112 | 21 | + | + | - | 167 | 28 | - | - | - |
| 3 | 7 | + | - | - | 58 | 14 | + | + | - | 113 | 21 | - | - | - | 168 | 28 | - | - | - |
| 4 | 7 | - | - | - | 59 | 14 | - | - | - | 114 | 21 | - | - | - | 169 | 28 | - | - | - |
| 5 | 7 | + | - | - | 60 | 14 | + | - | - | 115 | 21 | + | + | - | 170 | 28 | - | - | - |
| 6 | 7 | + | - | - | 61 | 14 | + | + | + | 116 | 21 | - | - | - | 171 | 28 | - | - | - |
| 7 | 7 | - | - | - | 62 | 14 | - | - | - | 117 | 21 | - | - | - | 172 | 28 | - | - | - |
| 8 | 7 | + | - | - | 63 | 14 | - | - | - | 118 | 21 | - | - | - | 173 | 28 | - | - | - |
| 9 | 7 | - | - | - | 64 | 14 | + | + | - | 119 | 21 | - | - | - | 174 | 28 | - | - | - |
| 10 | 7 | + | + | - | 65 | 14 | - | - | - | 120 | 21 | - | - | - | 175 | 28 | - | - | - |
| 11 | 7 | - | - | - | 66 | 14 | - | - | - | 121 | 21 | - | + | - | 176 | 28 | - | - | - |
| 12 | 7 | + | - | - | 67 | 14 | - | - | - | 122 | 21 | - | - | - | 177 | 28 | - | - | - |
| 13 | 7 | + | - | - | 68 | 14 | + | - | - | 123 | 21 | + | - | - | 178 | 28 | - | - | - |
| 14 | 7 | + | - | - | 69 | 14 | - | - | - | 124 | 21 | - | - | - | 179 | 28 | - | - | - |
| 15 | 7 | + | + | - | 70 | 14 | + | + | + | 125 | 21 | + | + | - | 180 | 28 | - | - | - |
| 16 | 7 | + | - | - | 71 | 14 | - | - | - | 126 | 21 | - | - | - | 181 | 28 | - | - | - |
| 17 | 7 | + | + | - | 72 | 14 | - | - | - | 127 | 21 | + | - | - | 182 | 28 | + | + | + |
| 18 | 7 | + | - | - | 73 | 14 | - | + | - | 128 | 21 | + | - | - | 183 | 28 | + | + | - |
| 19 | 7 | + | - | - | 74 | 14 | + | - | - | 129 | 21 | - | - | - | 184 | 28 | - | - | - |
| 20 | 7 | - | - | - | 75 | 14 | - | - | - | 130 | 21 | + | + | - | 185 | 28 | - | - | - |
| 21 | 7 | + | - | - | 76 | 14 | - | - | - | 131 | 21 | - | - | - | 186 | 28 | - | - | - |
| 22 | 7 | + | - | - | 77 | 14 | + | + | - | 132 | 21 | - | - | - | 187 | 28 | - | - | - |
| 23 | 7 | + | - | - | 78 | 14 | - | + | - | 133 | 21 | + | - | - | 188 | 28 | - | - | - |
| 24 | 7 | + | - | - | 79 | 14 | + | - | - | 134 | 21 | - | - | - | 189 | 28 | - | - | - |
| 25 | 7 | + | - | - | 80 | 14 | - | - | - | 135 | 21 | - | - | - | 190 | 28 | - | - | - |
| 26 | 7 | + | - | - | 81 | 14 | - | + | - | 136 | 21 | - | - | - | 191 | 28 | - | - | - |
| 27 | 7 | + | - | - | 82 | 14 | + | + | - | 137 | 21 | - | - | - | 192 | 28 | - | - | - |
| 28 | 7 | + | - | - | 83 | 14 | - | - | - | 138 | 21 | - | - | - | 193 | 28 | - | - | - |
| 29 | 7 | + | - | - | 84 | 14 | - | - | - | 139 | 21 | - | - | - | 194 | 28 | - | - | - |
| 30 | 7 | + | - | - | 85 | 14 | + | - | - | 140 | 21 | - | - | - | 195 | 28 | - | - | - |
| 31 | 7 | + | - | - | 86 | 14 | - | - | - | 141 | 21 | - | - | - | 196 | 28 | - | - | - |
| 32 | 7 | - | - | - | 87 | 14 | - | + | - | 142 | 21 | - | - | - | 197 | 28 | + | + | + |
| 33 | 7 | + | + | - | 88 | 14 | + | + | - | 143 | 21 | - | - | - | 198 | 28 | - | - | - |
| 34 | 7 | + | + | - | 89 | 14 | - | - | - | 144 | 21 | - | - | - | 199 | 28 | - | - | - |
| 35 | 7 | + | + | - | 90 | 14 | - | - | - | 145 | 21 | - | - | - | 200 | 28 | - | - | - |
| 36 | 7 | + | + | - | 91 | 14 | - | - | - | 146 | 21 | - | - | - | 201 | 28 | - | - | - |
| 37 | 7 | + | + | - | 92 | 14 | - | - | - | 147 | 21 | - | - | - | 202 | 28 | - | - | - |
| 38 | 7 | + | + | - | 93 | 14 | + | + | + | 148 | 21 | - | - | - | 203 | 28 | - | - | - |
| 39 | 7 | + | + | - | 94 | 14 | - | - | - | 149 | 21 | - | - | - | 204 | 28 | - | - | - |
| 40 | 7 | + | + | + | 95 | 21 | + | + | - | 150 | 21 | + | - | - | 205 | 28 | - | - | - |
| 41 | 7 | + | - | - | 96 | 21 | + | + | - | 151 | 21 | - | - | - | 206 | 28 | - | - | - |
| 42 | 7 | + | - | - | 97 | 21 | + | + | + | 152 | 21 | + | - | - | 207 | 28 | - | - | - |
| 43 | 14 | - | - | - | 98 | 21 | + | - | - | 153 | 28 | - | - | - | 208 | 28 | - | - | - |
| 44 | 14 | - | - | - | 99 | 21 | - | - | - | 154 | 28 | - | - | - | 209 | 28 | - | - | - |
| 45 | 14 | - | - | - | 100 | 21 | + | + | + | 155 | 28 | - | - | - | 210 | 28 | - | - | - |
| 46 | 14 | + | + | + | 101 | 21 | - | - | - | 156 | 28 | - | - | - | 211 | 28 | - | - | - |
| 47 | 14 | + | + | - | 102 | 21 | - | - | - | 157 | 28 | - | - | - | 212 | 28 | - | - | - |
| 48 | 14 | - | - | - | 103 | 21 | - | - | - | 158 | 28 | - | - | - | 213 | 28 | - | - | - |
| 49 | 14 | - | + | - | 104 | 21 | + | + | - | 159 | 28 | - | - | - | 214 | 28 | - | - | - |
| 50 | 14 | - | - | - | 105 | 21 | - | - | - | 160 | 28 | - | - | - | 215 | 28 | - | - | - |
| 51 | 14 | + | - | - | 106 | 21 | + | + | + | 161 | 28 | + | - | - | 216 | 28 | - | - | - |
| 52 | 14 | - | - | - | 107 | 21 | - | - | - | 162 | 28 | - | - | - | 217 | 28 | - | - | - |
| 53 | 14 | + | + | - | 108 | 21 | - | - | - | 163 | 28 | - | - | - | 218 | 28 | - | - | - |
| 54 | 14 | - | - | - | 109 | 21 | + | + | - | 164 | 28 | - | - | - |  |  |  |  |  |
| 55 | 14 | - | - | - | 110 | 21 | - | - | - | 165 | 28 | - | - | - |  |  |  |  |  |
